# LeafSense: Leveraging Wi-Fi Signals for Monitoring Seasonal Changes †

**DOI:** 10.3390/s26216674

**Published:** 2026-10-22

**Authors:** Saeid Mehrdad, Alamin Mohammed, Aaron Striegel

**Affiliations:** Department of Computer Science and Engineering, University of Notre Dame, Notre Dame, IN 46556, USA; smehrdad@nd.edu (S.M.); amohamm2@nd.edu (A.M.)

**Keywords:** Wi-Fi sensing, environmental sensing, classification, Wi-Fi signals

## Abstract

Wi-Fi sensing has attracted growing interest due to the ubiquity of Wi-Fi signals and their promise as a low-cost sensing modality. Knowing when the leaves in a neighborhood have fallen is needed to schedule municipal leaf collection, which is difficult to plan with the means currently available to cities. In this paper, we introduce LeafSense, a system that detects the seasonal falling of leaves through variations in the received signal strength indicator (RSSI) of beacons broadcast by existing Wi-Fi access points (APs). The idea behind LeafSense is that the environment around a transmitter shapes how its signal propagates, and the presence or absence of leaves changes the received signal accordingly. To verify this, we built a sensing rig from commodity devices that monitors 11 Wi-Fi channels in the 2.4 and 5 GHz bands, and drove it through several neighborhoods over multiple seasons. A multi-step filtering pipeline removes vehicle-induced artifacts. Decision Tree and Random Forest classifiers distinguish the presence and absence of leaves with 81% to 94% accuracy, whereas the same classifiers trained on two weeks with unchanged foliage perform near chance. We further show that the sensing rig can be simplified, as two 5 GHz channels are sufficient, covering more than 90% of the surveyed area with 81% to 97% accuracy, reducing the rig to a single Raspberry Pi with two Wi-Fi adapters. Finally, we show that the results are robust to small variations in the vehicle’s route and that AP density is sufficient for reliable sensing.

## 1. Introduction

While Wi-Fi signals have been used primarily for providing connectivity, recent advances have demonstrated Wi-Fi signals to be sensitive enough to identify and sense environmental changes [1,2]. This capability makes Wi-Fi signals potentially an effective tool for sensing in a wide range of scenarios, such as gesture recognition [3,4,5], activity recognition [6,7], healthcare applications [8,9,10], crowd counting [11,12], and localization [13,14,15]. The main principle behind Wi-Fi sensing is that, because of the interactions between the Wi-Fi signals with the objects in the same environment, Wi-Fi signals will vary with changes in the environment. Through analysis of the Wi-Fi signal variation, valuable insights about the surrounding physical environment can be obtained [16].

Notably, many of the aforementioned applications were designed for usage indoors and oriented at human sensing. In this work, we examine the extent to which Wi-Fi sensing can address a specific seasonal issue, specifically whether or not leaves have fallen in a suburban environment, for the purposes of improved leaf collection. For many cities with a large number of trees that drop their leaves in the colder months, the leaf debris can be problematic due to either leaf litter [17] or particle pollution generated from leaf burning. Many such cities offer leaf pickup services, either for free or for a subsidized fee. Unfortunately, it can be quite difficult to determine where to send the leaf pickup trucks, as variables such as the tree type and weather may result in significant variations, both within a neighborhood and from season to season. Further, remote sensing approaches such as satellite imaging or unmanned aerial vehicles can be prohibitively expensive [18,19,20]. Satellite-based phenology monitoring is further constrained by the trade-off between spatial and temporal resolution and by cloud contamination, which hinders detection of fine-scale responses across heterogeneous landscapes [21]. Deep learning applied to aerial imagery has been used to map urban street trees across multi-year periods [22], capturing tree presence rather than seasonal leaf state. While more recent systems combine unmanned aerial vehicles with ground-fixed cameras to resolve canopy phenology at fine spatial scales [23], they still require dedicated instrumentation to be deployed and maintained at each site. Existing wireless infrastructure has likewise been repurposed for environmental monitoring, with urban rainfall inferred from link attenuation in community mesh networks [24] and from the received signal strength of LTE base stations without additional hardware [25]. The premise of this paper is to use Wi-Fi signals to inexpensively bridge this gap.

**Figure 1:**
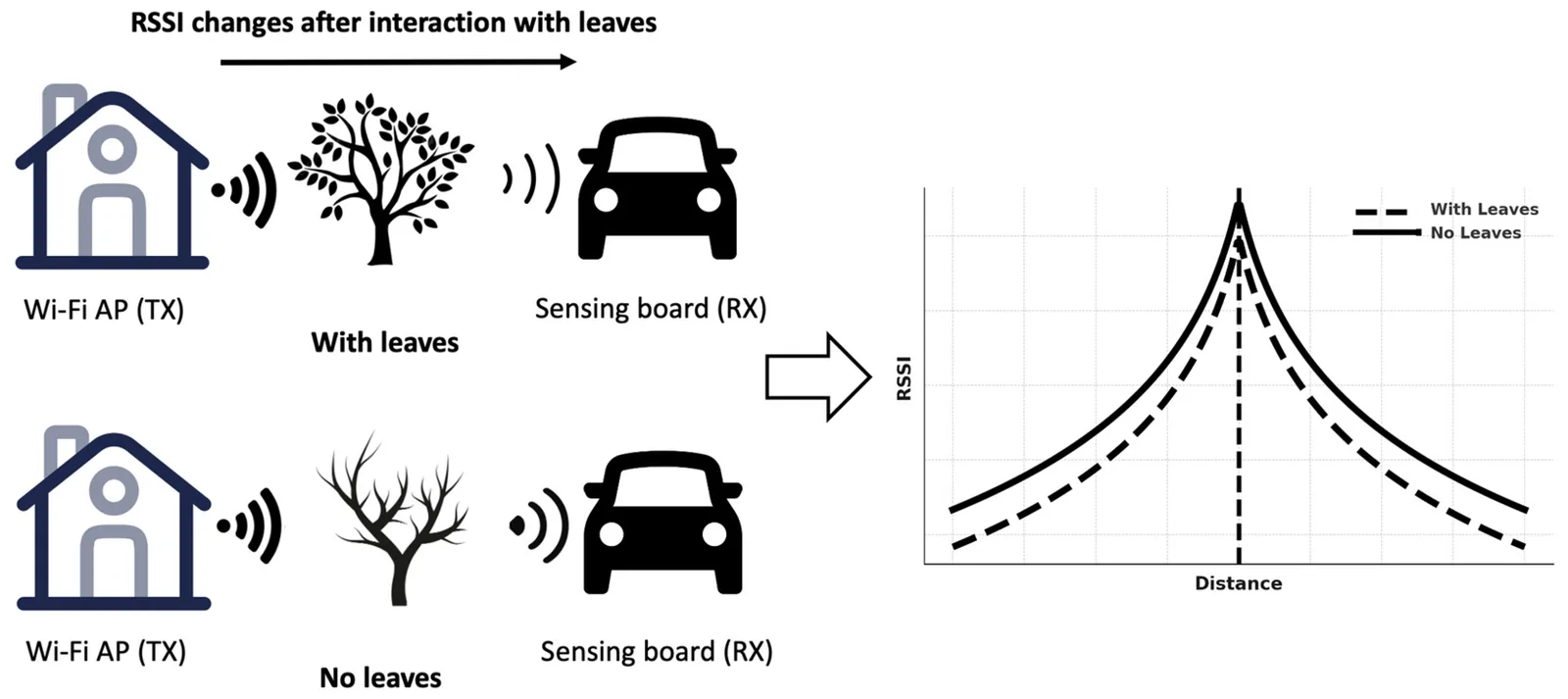
RSSI changes in presence and absence of leaves. The curves are a conceptual illustration of the expected behavior and do not represent measured data.

In this paper, we introduce LeafSense, a novel approach that deploys Wi-Fi signals—to the best of our knowledge—for monitoring the timing of significant leaf fall in an urban environment. The intuition behind LeafSense, as shown in Figure 1, is that leaves when present lead to distinct impacts on Wi-Fi signals. Leaves, branches and trunks absorb and scatter radio waves, and the resulting excess attenuation grows with the depth of foliage that the signal traverses and with the carrier frequency. Empirical models such as the modified exponential decay model of Weissberger [26] predict attenuation rates of a fraction of a dB to a few dB per meter of foliage at these frequencies. Measurements at 2.45 and 5.25 GHz have shown considerably deeper fading through foliated than through non-foliated deciduous trees [27], and campaigns through tree canopies have confirmed that the attenuation of trees in leaf is consistently higher than that of the same trees out of leaf [28,29]. At 2.4 GHz, where the wavelength is comparable to the size of the leaves, the additional loss due to foliage is more moderate [30]. In a residential street, the beacons of an AP located inside a house reach a passing vehicle through the trees in the front yard, so the seasonal change in foliage is expected to appear as a measurable change in the RSSI observed from the street. We show through experimental studies that variations in RSSI can be effectively captured on commodity Wi-Fi devices, processed through streamlined data filtering, and used to accurately classify the presence or lack thereof of leaves on trees. Furthermore, we demonstrate these capabilities across seasonal and neighborhood variations to show that the approach is robust and repeatable while operating only on a minimal subset of Wi-Fi channels.

The main contributions are summarized as follows:

- *Robust sensing dataset:* We conducted an extensive, long-term Wi-Fi data collection across multiple seasons and neighborhoods. Data was gathered by mimicking the natural patterns of city vehicles and utilizing a robust sensing rig equipped with 13 Raspberry Pi 4s capable of monitoring 11 distinct Wi-Fi channels via external USB Wi-Fi adapters, a dedicated Raspberry Pi for GPS, and a dedicated Raspberry Pi for orchestration. In our study, we captured over 339,610 RSSI samples from more than 3840 unique access points gathered from beacon frames broadcast from said access points. The dataset and sensing rig will be made available as open source. Proper anonymization includes location, BSSID, and SSID obfuscation through hashing and privacy-sensitive location transformations. This matters because databases linking BSSIDs to locations have been shown to enable device geolocation and tracking at global scale [31]. No data frames were saved as part of the collection effort.
- *Effective data processing:* We implemented a multi-step data cleaning and processing pipeline to address the challenges of collecting data with a moving vehicle. By filtering out redundant data caused by factors such as obstacles, traffic, and intermittent stops, we refined the dataset to yield roughly 130,033 actionable RSSI sample points and 1972 unique access points that in turn could be leveraged by our classification process;
- *Wi-Fi as a sensor for leaf detection:* We show that Wi-Fi access points can serve as a type of “sensor device” to indicate leaf presence or absence by leveraging RSSI variations as indicators. Across different locations and months, we obtained classification accuracy using Decision Trees and Random Forests to yield ranges from 81% to 94%, demonstrating the potential of RSSI to function as a reliable indicator for leaf status monitoring;
- *Two channels are enough:* By analyzing the coverage of each Wi-Fi channel in the data collection neighborhood, we evaluated the performance of 2.4 and 5 GHz channels for our sensing objectives and explored cost reduction for the sensing rig. We show with our exploration that even two channels in the 5 GHz band can achieve good results for classification, making LeafSense achievable with a small footprint (single device—two Wi-Fi adapters);
- *Robustness to variability:* Finally, we evaluated the robustness of our data collection approach by examining its resilience to variations in data gathering paths and assessing the density of the access points in different city locations. We show that regardless of the path taken through a neighborhood that the classification results remain excellent. Further, we show that access point density fares remarkably well even in areas with sparser density, e.g., suburban neighborhoods. Moreover, in contrast to a vision-based solution (e.g., inexpensive IoT camera), LeafSense in its minimal form (two channels) has little required processing, calibration, or effort for deployment.

This article is a revised and expanded version of a paper entitled “Leveraging Wi-Fi Signals for Monitoring Seasonal Changes” [32], which was presented at the 2026 35th International Conference on Computer Communications and Networks (ICCCN), Honolulu, HI, USA, 27–30 July 2026. Compared with the conference version, this article extends the introduction and the review of related work with the recent literature on environmental monitoring from imagery and from existing wireless infrastructure as well as with the background on CSI- and RSSI-based sensing (Section 1 and Section 2), describes the configuration of the sensing rig (Section 3.2), and adds two new sets of results: the analysis of channel prevalence that leads to the optimized two-channel sensing rig and the resulting classification accuracy (Section 5.2), and the dynamic channel-switching design for city blocks together with the measurement of the channel-switching time (Section 5.3). The remaining sections have been revised and expanded with additional detail on the data processing pipeline.

## 2. Background and Related Work

Wi-Fi sensing has recently drawn significant attention in a variety of studies and applications. In this section, we review some of the existing work from two aspects, Channel State Information (CSI)-based Wi-Fi sensing [33,34,35] and RSSI-based Wi-Fi sensing [36,37]. We first begin with a brief overview of CSI and RSSI and then continue with discussing related work.

To start, Channel State Information or CSI represents detailed data on how wireless signals propagate from a transmitter to a receiver, capturing the impact of the environment on signal transmission [33]. The Channel State Information contains information such as time delay, amplitude attenuation, and phase shift, which can then be employed to analyze how the signal undergoes reflection, refraction, and scattering along multiple paths [34]. Although the recently ratified IEEE 802.11bf amendment [38] standardizes sensing measurement and CSI reporting procedures, such support is not yet present on commodity devices, where access to CSI still requires specific hardware coupled with particular drivers or the capture of raw signal.

Similarly, the Received Signal Strength Indicator (RSSI) can fluctuate based on the characteristics of the surrounding environment and distance to various objects. Whereas CSI offers a much richer corpus of data, RSSI is much more commonly available on many Wi-Fi devices. Notably, the availability of RSSI from access point beacons that are issued periodically (roughly ten times per second) is often a frequently used mechanism for gathering RSSI. However, the granularity of RSSI can vary significantly. For example, typical system call interfaces to Wi-Fi to such information may only offer averaged RSSI and may further restrict views of relevant protocol information (e.g., hardware addresses of access points such as on iOS or macOS). Such restrictions are motivated by privacy, as RSSI and access point identifiers can be used to infer the locations a person frequents [39]. For the purposes of this paper, we work with per-packet RSSI as can be gathered using a commodity device such as the Raspberry Pi in conjunction with an external Wi-Fi USB adapter. RSSI also has well-known limitations. Being a single value per packet, it aggregates the contributions of all propagation paths, so it is sensitive to multipath fading and to shadowing by nearby objects. It depends on the transmit power of the AP, which is unknown and may differ between devices, and it depends on the gain and calibration of the receiving hardware, so that two adapters may report different values for the same signal [36,37]. For these reasons, RSSI-based sensing generally relies on aggregate statistics over many packets rather than on individual readings. In this work, we mitigate these effects by working with per-second averages, by comparing APs across months with the same receiver on each channel, and by filtering out the samples captured while the vehicle was stationary or on the far side of the street (Section 4.3).

Next, we continue by examining two application areas where Wi-Fi sensing has been employed, namely material sensing [40,41,42,43] and event sensing [44,45,46]. Material sensing primarily focuses on structural and object monitoring as well as biological and agriculture sensing. Tan et al. [16] proposed FruitSense, employing Wi-Fi signals to extract the multipath-independent signal components, represented as CSI, to analyze the physiological compounds of the fruit associated with fruit ripening, enabling fruit ripeness detection. By employing the refraction of Wi-Fi signals and Multiple-Input-Multiple-Output (MIMO) technology, Wang et al. [47] introduced a grain moisture sensing system, GrainSense. It correlates grain moisture with the phase difference between refracted Wi-Fi signals, mitigates multipath interference in indoor environments by utilizing Wi-Fi beamforming, and introduces the Wi-Fi CSI beamforming ratio to remove frequency and phase biases. Utilizing CSI, Wang et al. [48] proposed a method for detecting dangerous objects inside luggage. By reconstructing the complex CSI of signals reflected by the object, this work identifies the type of dangerous material and determines the risk level associated with the object. Wi-Tar [49] is another Wi-Fi-based object detection system utilizing the quotient of CSI as the base signal and assessing the differences between materials using the reconstructed CSI complex. Ren et al. [50] developed LiquidSense, a Wi-Fi-based system for detecting liquid levels in containers, which leverages home Wi-Fi networks and a low-cost transducer to measure resonance frequency changes associated with liquid level changes. Salman et al. [51] related RSSI to soil water content in laboratory and field experiments, reporting strong linear correlations with *R*^2^ between 0.92 and 0.99.

Event sensing generally focuses on monitoring the state of the sensing object. In recent years, Wi-Fi-based event sensing has primarily aimed at human-centric events and static event detection. Tian et al. [36] proposed an RSSI-based event sensing, RSSI-WSDE, which processes original RSSI time series by leveraging variable-length sliding windows and statistical methods to differentiate dynamic events from static environment. An indoor RSSI-based crowd estimation system, utilizing the signal absorption rate of the human body to Radio Frequency signals, is presented by Taha et al. [52]. The estimation is implemented applying a pre-trained Convolutional Neural Network model. Bianchi et al. [53] proposed an indoor RSSI-based room-level localization system leveraging a threshold algorithm derived from receiver operating characteristic analysis. Based on the users’ interactions with home system devices deployed throughout the house, their location is estimated. Booranawong et al. [54] introduced a Wi-Fi-based device-free human detection and tracking system using RSSI. To accurately track human activity, this system utilizes a predefined threshold and zone selection method. They later developed an adaptive filtering method that automatically filters inputs with high variation, enhancing detection accuracy while reducing computational complexity [55]. More recently, Wang et al. [56] showed that RSSI alone can support passive tracking on commodity hardware, presenting it as a practical alternative where CSI access is restricted.

Our work differs in many key aspects from these studies. First, many of the previous works were conducted in highly controlled experimental environments, and as a consequence, their results, especially those based on CSI sensing, can be sensitive to environmental changes, which may impact the outcomes [57]. Second, while the aforementioned studies primarily focus on indoor event sensing, our work addresses outdoor event sensing. In recent years, vehicle movement sensing [58,59,60,61] has also seen considerable interest. However, such works often require additional sensors and devices to collect augmented data such as velocity. In contrast, our approach does not rely on specific environmental setups or assumptions and utilizes robust data processing techniques to monitor seasonal changes effectively.

To the best of our knowledge, the closest related efforts are, on the one hand, the vegetation attenuation measurements of the propagation literature [26,27,28,29,30], which quantify the excess loss of a dedicated link through foliage under a controlled geometry, and, on the other hand, the agricultural Wi-Fi sensing systems discussed above [16,47,51], which target a single object with a purpose-built transmitter–receiver pair. LeafSense differs from both in that it relies only on the beacons of existing APs, received opportunistically from a moving vehicle, and in that it uses their seasonal RSSI variation at the scale of a neighborhood. Closer to our setting, Lacan and McBride [62] measured the attenuation of municipal Wi-Fi links by individual street trees and found losses between 2 and 19 dB that were explained by tree size, canopy depth and leaf type, whereas the presence of leaves did not emerge as an explanatory variable in their static measurements. LeafSense does not compare different trees with each other. It compares the same APs across seasons, which isolates the change due to foliage from the differences between trees and links. While the underlying attenuation effect is well documented, to the best of our knowledge no previous work has used ambient Wi-Fi signals to monitor seasonal changes in foliage, and in this sense LeafSense is the first approach of its kind.

## 3. Experimental Platform

As described in the introduction, a significant corpus of data was gathered for the purpose of evaluating the extent to which RSSI can be leveraged to detect the presence or absence of leaves. Given that the emergence and fall of leaves is a seasonal process, this involved gathering data over a multi-seasonal period including both fall observing the leaves going from present to absent as well as the spring going from absent to present. To that end, we created a sensing rig drawn from commodity hardware consisting of a set of Raspberry Pi nodes, external USB Wi-Fi adapters, and a GPS sensor.

### 3.1. Sensing Rig Hardware

**Figure 2:**
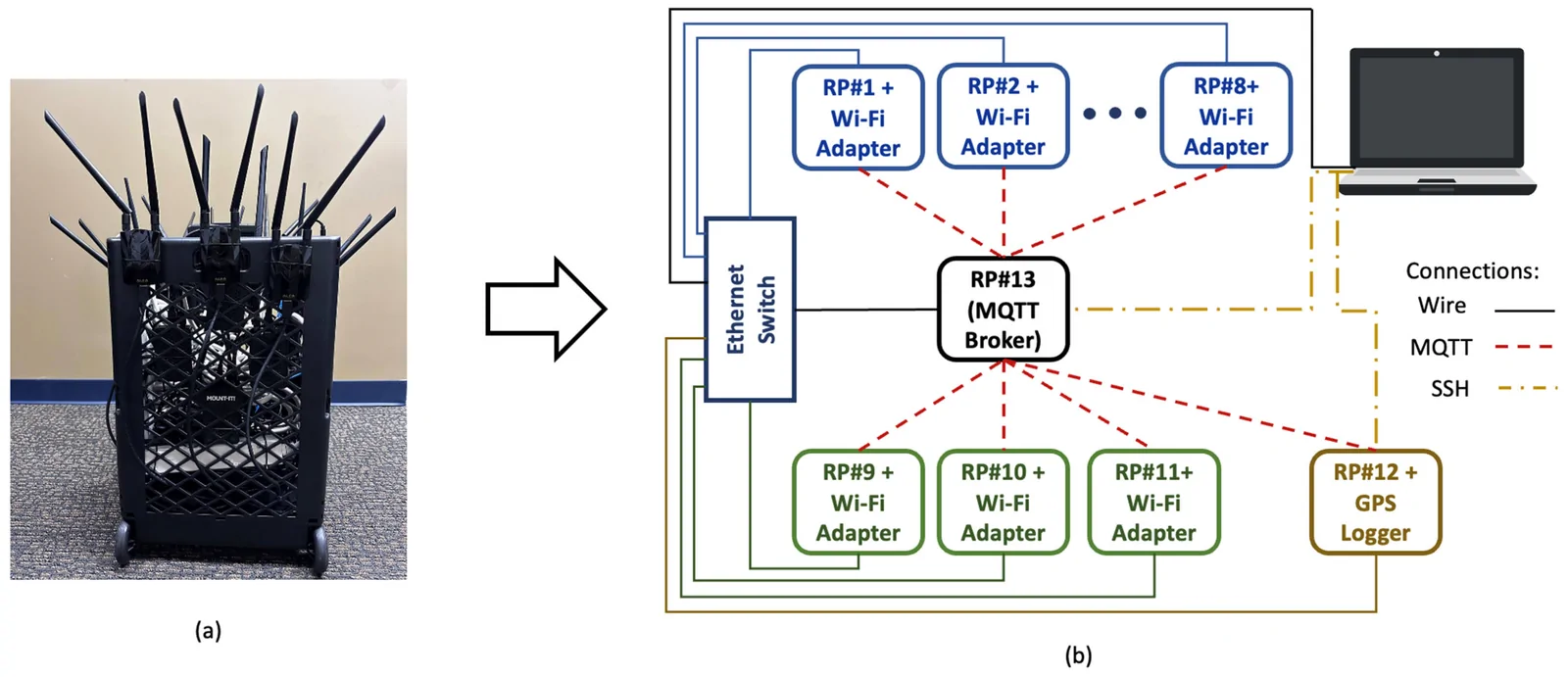
Hardware for data gathering including (**a**) picture of the sensing rig and (**b**) component diagram of the sensing rig. In (**b**), the dots indicate the omitted nodes RP#3 to RP#7, and the line styles denote the wired, MQTT and SSH connections as given in the legend.

Figure 2 illustrates the design of the sensing rig, which includes 13 Raspberry Pis, each designated for a specific task. These include 8 Raspberry Pis capturing beacons from popular Wi-Fi channels within the 5 GHz band—channels 36, 40, 44, 48, 149, 153, 157, and 161—and 3 Raspberry Pis monitoring channels 1, 6, and 11 within 2.4 GHz band. Each of these Raspberry Pis is set up to monitor a unique channel and equipped with a USB Wi-Fi adapter that is capable of operating in monitor mode under the IEEE 802.11 protocol. In order to boost the signal reception and improve the range of packet capture, the adapters are enhanced with external antennas. All nodes use the same adapter model, the ALFA AWUS036ACH, a dual-band IEEE 802.11ac USB adapter based on the Realtek RTL8812AU chipset, each fitted with two detachable 5 dBi omnidirectional dual-band antennas (RP-SMA). The adapters are placed in monitor mode and tuned to their assigned channel with the Linux iw and iwconfig utilities, and the beacon frames are captured with tshark. The RSSI of each frame is read from the radiotap header of the capture. No per-adapter calibration of the reported RSSI was performed. Instead, each channel was monitored by the same Raspberry Pi and adapter in all data collection runs, so that the comparison between months on a given channel is always made with the same receiver and any constant offset of that receiver is removed. Since the absolute RSSI reported by different adapters may nevertheless differ by several dB, the mean RSSI of each channel is included as a feature in the classification (Section 5.1) and the channels are selected per location (Section 5.2). In terms of sensing parameters, each node reports the RSSI of every received beacon frame in dBm with a resolution of 1 dB, at the beacon rate of the APs, on its fixed channel. The rig is entirely passive, as it only receives beacons and never transmits. In addition, one Raspberry Pi is augmented with a BerryGPS-IMU v3 module to obtain the geographical locations of the captured packets, integrating GPS data with Wi-Fi signal analysis for spatial mapping of signal strength. The final Raspberry Pi serves as the head unit, coordinating the data collection across all channels. All Raspberry Pis are interconnected via Ethernet cables connected through an Ethernet switch, ensuring a robust connection amongst all components.

### 3.2. Configuration

Upon system initialization, each Raspberry Pi on the rig is programmed to enter monitor mode with a filter to capture only access point beacons. This step allows the Raspberry Pis to capture the relevant Wi-Fi packets on their configured channels without the need to connect to a network. In order to streamline the interaction between Raspberry Pis, Message Queuing Telemetry Transport (MQTT) is utilized as the communication protocol for the system. The head Raspberry Pi is established as the MQTT broker, serving as the central hub for data transmission. The client Pis are programmed to subscribe to a time synchronization topic as well as publish their collected data packets to a specific MQTT topic, to which the head Pi has subscribed. The time is set manually at the beginning of the data collection in the broker and propagated to all Raspberry Pis through MQTT messages. In this way, it is ensured that packet capturing is synchronized across the monitoring nodes, facilitating the storage and organization of incoming data streams for subsequent retrieval and analysis. Additionally, through the remote access provided by Secure Shell (SSH), commands are executed directly from a laptop, enabling the initiation, real-time monitoring, and completion of the packet capturing process. Finally, the captured data is transferred from the head Raspberry Pi to the laptop using a File Transfer Protocol (FTP) application.

## 4. Methodology

In this section, we describe our collection methodology including the data collection approach, data preparation, and data processing. We begin first by describing the multi-seasonal data collection approach.

### 4.1. Data Collection Approach

**Figure 3:**
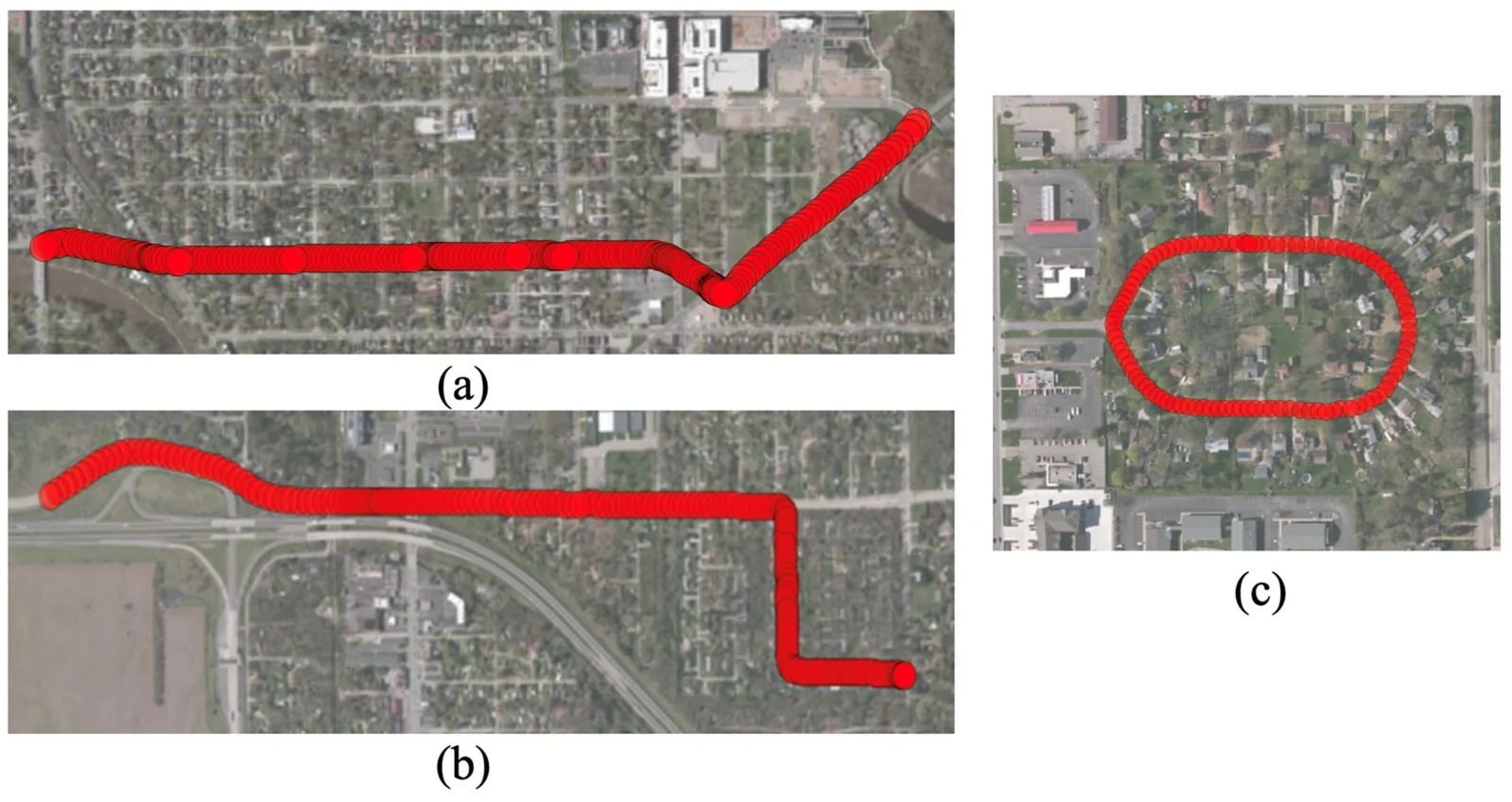
Areas of data collection. (**a**) Place A: the area covered during the February and May. (**b**) Place B: the area covered during the September and February. (**c**) Place C: the area, considered as a representative city block, covered during the peak leaf-falling period in October and November. The red dots mark the route driven by the vehicle, as recorded by the GPS logger of the sensing rig.

Utilizing the capabilities of our sensing rig, we executed a multi-phased data collection across a city in the Midwest of the US, aimed at enhancing our understanding of Wi-Fi signal variability in relation to seasonal leaf density. Deploying the sensing rig aboard a vehicle, we traversed various locations to collect data (Figure 3). Initially, we collected data during different seasons—summer, fall, and winter—along with GPS data to investigate how the presence or absence of foliage impacts Wi-Fi signal strength. Furthermore, during the peak leaf-falling period from 1 October to 14 November, focused data collection was conducted at a specific city block, considered as a representative model for urban areas. This effort aimed to establish a Wi-Fi RSSI signature for each block, monitoring variations in Wi-Fi data due to leaf fall to develop a leaf collection schedule based on the observed variations. This analysis serves as a prototype for broader urban areas, suggesting potential applications of our findings. Finally, we carried out extended drives through various city sectors to gather comprehensive data. This phase focused on statistically analyzing the availability of APs and the number of beacon frames captured, providing insights into the Wi-Fi landscape across different areas.

It is worth noting that for all data collection runs, we chose residential areas, specifically neighborhoods with high tree density, to ensure both a sufficient presence of Wi-Fi signals and an abundance of foliage for robust data collection. Additionally, we ensured that these residential areas remained unchanged throughout our data collection period, thereby minimizing any environmental factors, apart from the presence of foliage, that could potentially influence signal strength variations. This included avoiding locations where significant alterations, such as new construction or infrastructure changes, occurred during our data collection periods. This approach allowed us to analyze Wi-Fi signals in environments where the presence of foliage could noticeably impact signal strength. These areas are shown in Figure 3. In addition, since RSSI values are influenced by the distance between the transmitter and receiver, we tried to ensure consistency in our data collection by maintaining a steady speed of approximately 20–30 miles per hour and positioning the rig in the same location within the car for all our data collection runs. Each run lasted approximately 5 to 10 min. The trees along the routes are the deciduous street and yard trees typical of residential neighborhoods in the Midwestern United States. No particular species was targeted, since the objective of LeafSense is the leaf status of a neighborhood as a whole, as relevant for leaf collection, rather than the identification of individual trees.

### 4.2. Data Preparation

The raw data captured during our collection runs includes beacon frames across 2.4 GHz and 5 GHz Wi-Fi channels, stored in Packet Capture (pcap) format, which is the standard file format for capturing network traffic. For ease of analysis, these pcap files were converted into Comma-Separated Values (CSV) format. Each CSV file contains a header with the following fields:

- frame.time: timestamp;
- wlan.sa: sender MAC address (Source Address—SA);
- wlan.da: recipient MAC address (Destination Address—DA);
- wlan.bssid: Basic Service Set Identifier (BSSID);
- radiotap.dbm_antsignal (The data as recorded comes from the radiotap header from pcap and can result in spurious values (e.g., positive values for RSSI). Through our processing algorithm as described later, these spurious data values are handled appropriately. For an accurate representation of the raw data, our later graphs do include those values.): Received Signal Strength Indicator (RSSI).

Then, a data cleansing process was implemented to address the issue of missing data by removing entries with incomplete values. For privacy considerations, we filtered BSSIDs associated with broadcast addresses ‘ff:ff:ff:ff:ff:ff’, since some BSSIDs may mask their MAC addresses. In addition, since a beacon’s destination address is always ’ff:ff:ff:ff:ff:ff’, we filtered the rows for which SA and BSSID do not have the same MAC address in order to keep only the frames from the real and reliable data. The RSSI, as reported by the adapter for each captured beacon, is the only signal indicator used in this work. CSI is not available on the commodity adapters used (Section 2), and no other measurement is required by the approach.

### 4.3. Data Processing

Due to several factors, including the speed of the vehicle, intermittent stops, and varying distances to APs, the raw data requires thorough filtering and processing to accurately reflect the underlying behavior of the RSSI values. To achieve this, we implemented a multi-step data filtering process described in the next sub-sections.

#### 4.3.1. Selection of Optimal APs

In the first step, in order to ensure that our analysis is based on reliable and valid data, it is essential to identify and select well-placed APs with sufficient data points to accurately reflect their Wi-Fi signal strength. However, for some APs, due to factors such as the distance of these APs from our vehicle and the speed at which we were driving, only a limited number of samples were collected, which do not provide meaningful or robust information about their signal strength. For this reason, the dataset needs to be filtered to exclude these APs, retaining only those with adequate data points. Several studies address the problem of appropriate sample size for APs and AP selection [60,63,64]. In some of these studies, around 30 data samples are suggested as an adequate number of sample points that can represent the characteristics of the RSSI of an AP. Although the assumptions and conditions under which data were collected in these studies differ from ours, we observed that when the number of sample points is larger than 30, the RSSI sample points follow the log-normal shadowing model, typically represented as [65]:

(1) $$\mathrm{RSS}I_{l}=\mathrm{RSS}I_{r}-10n\log_{10}\left(\frac{d_{l}}{d_{r}}\right)+X_{\sigma }$$

where *RSSI_l_* and *RSSI_r_* are the measured RSSI values at *d_l_* and the reference distance *d_r_*, respectively. The variable *n* denotes the path-loss exponent, which is indicative of the propagation environment. *d_l_* represents the distance between the receiver and AP, and *X_σ_* denotes a zero mean Gaussian random variable with a standard deviation *σ*.

**Figure 4:**
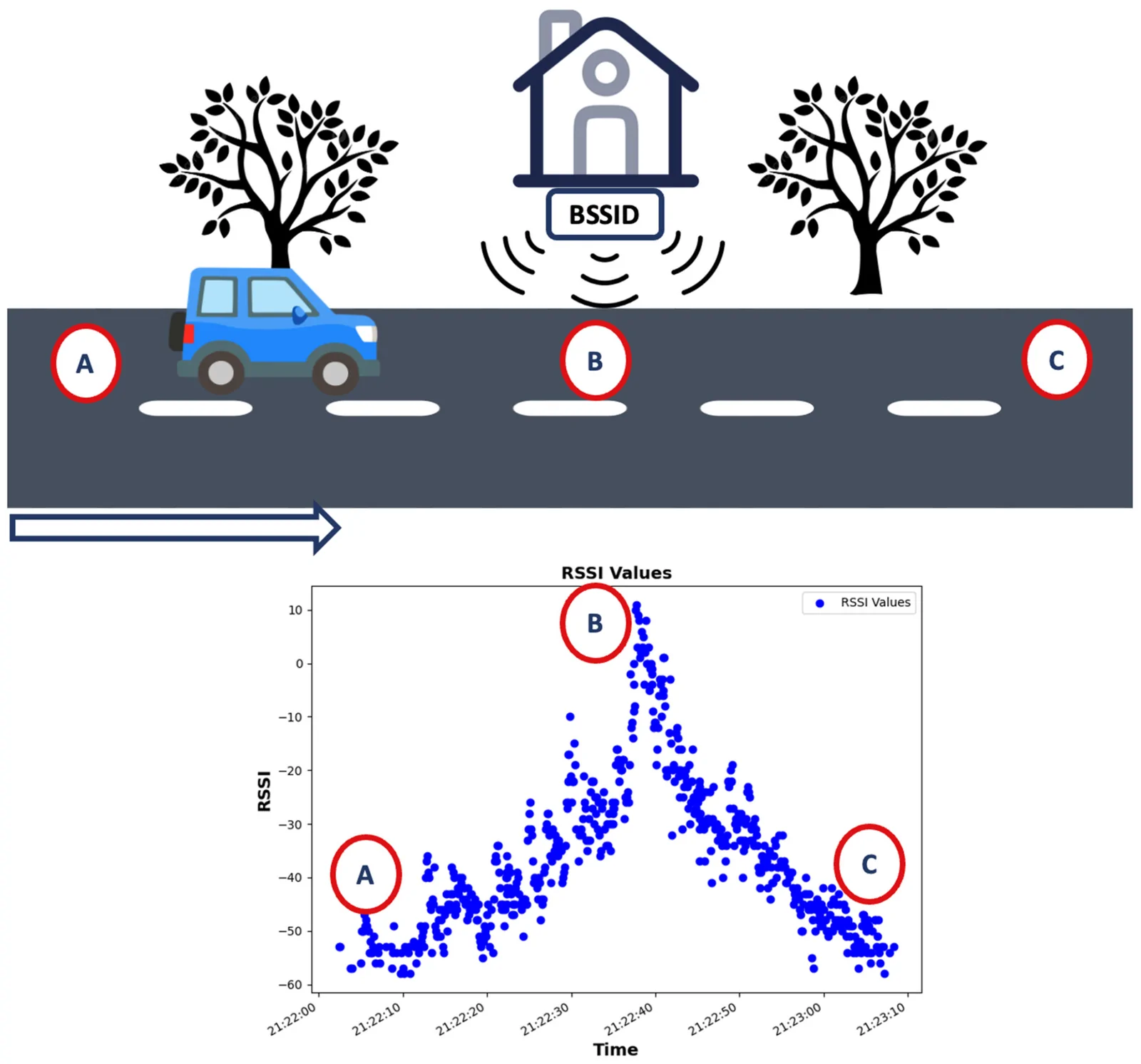
RSSI values captured while driving along a street. The plot below shows the corresponding RSSI variations as the vehicle moves closer to and farther from the BSSID, with peak signal strength at point B, where the vehicle was closest to the BSSID. Points A, B and C mark the positions along the route at which the vehicle is far from the AP, closest to it, and far from it again, and the arrow indicates the driving direction.

Based on this model, the RSSI increases as the vehicle approaches the AP and decreases as it moves farther away, reflecting the effect of path loss and log-normal shadowing on signal attenuation. Figure 4 illustrates the process of capturing RSSI values while driving along a street near an AP. This figure demonstrates the corresponding variations in RSSI as the vehicle moves from point A to point B, where it reaches peak signal strength when closest to the BSSID, and then decreases from point B to point C as the vehicle moves away from the AP. This behavior clearly shows how distance from the AP affects RSSI readings.

By selecting APs with more than 30 data samples, we ensure that the APs were located within our data collection path, and the vehicle passed close to these APs, capturing a full range of RSSI variations as it moved towards and then away from them based on log-normal shadowing model. This approach guarantees that the captured RSSI values are from reliable APs within the vehicle’s proximity, rather than distant APs that were not meaningfully captured by the sensing device.

#### 4.3.2. Filtering of RSSI Values

**Figure 5:**
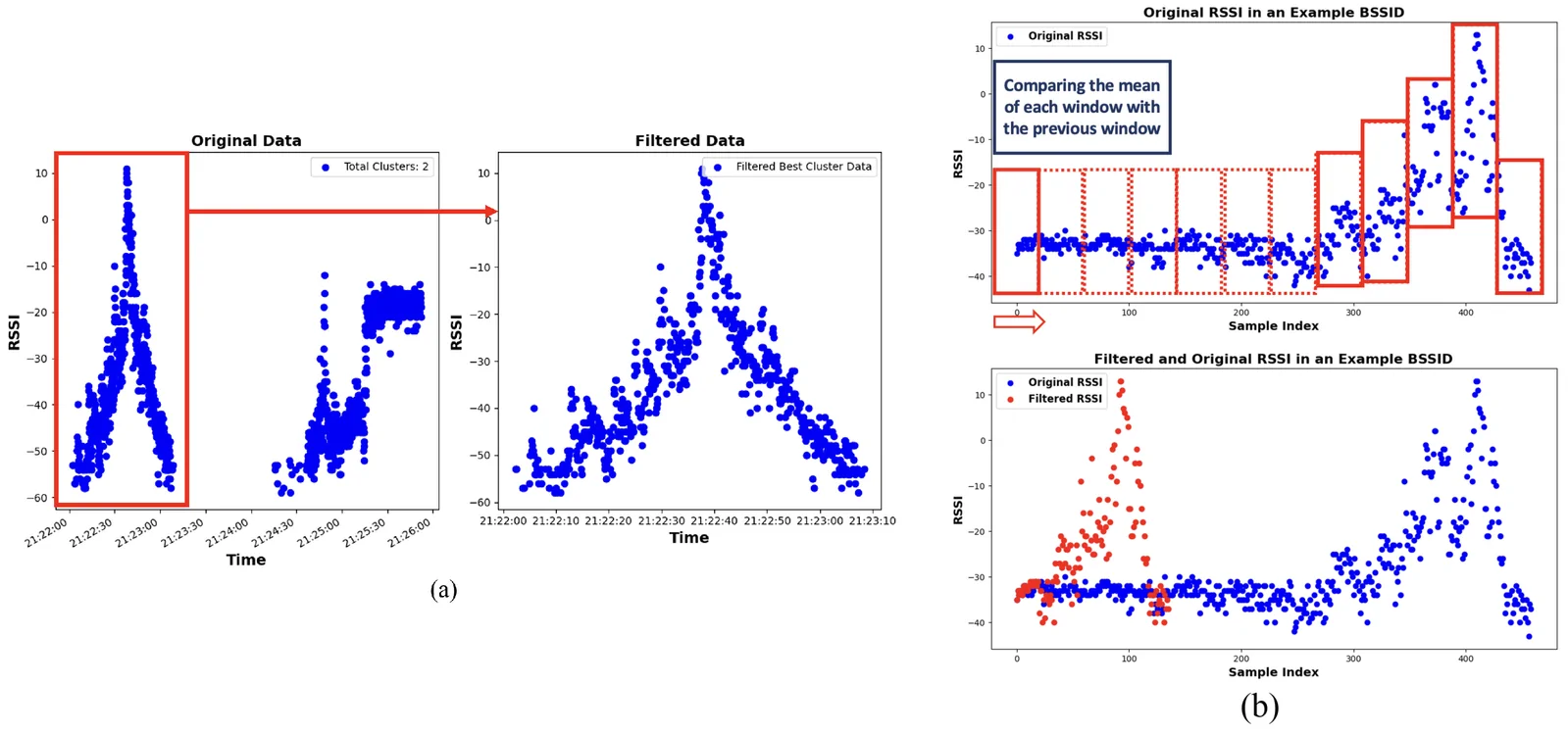
Filtering applied to the captured RSSI samples. (**a**) Filtering of the RSSI samples captured from repeated passes: the red frame marks the pass (cluster) retained by DBSCAN, which is shown on the right after filtering. (**b**) Window filtering: the mean RSSI of each one-second window (red frames) is compared with that of the previous window; dotted frames mark the windows whose change is below 1 dB, which are discarded, and the lower plot shows the retained samples in red.

In the second step, the RSSI values are filtered to remove noise and ensure that the processed data provides an accurate representation of the signal strength variations. During our data collection runs, we might pass certain locations more than once. For instance, we might drive down one side of a street and then return along the opposite side. In such scenarios, the RSSI values for the APs in those areas were captured multiple times at various time intervals as shown in the original data in Figure 5a. To ensure consistency in our dataset and minimize interference, we implemented a filtering process to retain only the RSSI values captured while driving on the side of the street closest to the APs. This method ensures uniformity in the dataset, enhancing the validity of comparisons across different months. Additionally, by focusing on the side of the street nearest to the APs, we reduce potential interference from factors such as passing vehicles.

To address this, we employed Density-Based Spatial Clustering of Applications with Noise (DBSCAN), a clustering algorithm that groups together data points that are closely packed and marks outliers as noise [66]. DBSCAN clusters RSSI values according to their spatial and temporal proximity. Since, based on Equation (1), RSSI decreases with distance, we selected the cluster with the highest mean among the top 10% of their RSSI values, ensuring that the retained data corresponds to the moments when the vehicle was closest to the APs. DBSCAN is a particularly good choice for our purpose, since it can detect clusters of varying density without requiring a predefined cluster number. It is essential for our dynamic driving conditions, in which the number of passes made by each AP may differ. Moreover, when collecting RSSI data, noise is inevitable. DBSCAN is effective at managing noise by identifying and removing outliers, ensuring that only the most useful data points are kept for analysis [67,68]. This filtering approach is depicted in Figure 5a.

DBSCAN is applied separately to the samples of each BSSID that passed the previous step. The clustering is performed on a single dimension, the capture time of the samples expressed in seconds, so that no scaling is required. Since the vehicle moves at a nearly constant speed, proximity in time corresponds to proximity in space along the route. We use ε = 10 s and min_samples = 50, i.e., a sample is a core point when at least 50 samples were captured within 10 s of it, which, at the beacon rate of roughly ten frames per second, corresponds to the AP being received continuously during that interval. With these values, samples captured during one pass along the AP form a single cluster, whereas passes separated by minutes form distinct clusters, and isolated samples are marked as noise. For a BSSID with a single cluster, all of its samples are kept. For a BSSID with several clusters, only the cluster whose top 10% RSSI values have the highest mean is retained, since it corresponds to the pass on the side of the street closest to the AP. The same parameter values are used for all locations, months and channels.

In this study, the average RSSI values of different APs collected across different months are compared to assess the existence of the leaves on the trees. Therefore, for a reliable comparison, it is crucial to only consider the RSSI values that follow the variation pattern described by Equation (1)—i.e., values that increase as the vehicle approaches the AP and decrease as it moves away. This is why it is important to remove data points that might skew the average or make it unreliable.

One such challenge arises when the vehicle is stationary. As illustrated in Figure 4, if the vehicle remains stationary between points A and C, it will collect multiple RSSI samples with similar values due to the constant distance from the AP, potentially skewing the calculated average for that AP. Figure 5b further demonstrates the impact of stationary data on overall RSSI measurements. Given that the sensing rig will be mounted on a vehicle, such as a garbage collection truck, which frequently makes stops, addressing this issue is crucial.

To address this, only the data collected while the vehicle is in motion needs to be considered. For this purpose, we apply a modified form of moving average filtering [69,70,71]. Based on the speed of the vehicle and the number of samples collected per second, we calculate the average RSSI for each second. Initially, if the average RSSI between consecutive seconds increases by at least 1 dB, this indicates that the vehicle is moving toward the AP. Once the maximum average RSSI is reached, the process reverses, and we retain only the data where the average RSSI decreases by at least 1 dB per second, indicating that the vehicle is moving away from the AP. Conversely, if consecutive seconds show a change of less than 1 dB, it suggests the vehicle is stationary, and these samples are discarded as they do not provide meaningful variations in signal strength. Following this approach, only the data reflecting changes in RSSI of at least 1 dB per second during movement are retained, ensuring the analysis is based on dynamic, relevant signal data. This filtering process is illustrated in Figure 5b.

The threshold of 1 dB per second was chosen from the expected rate of change in the RSSI while the vehicle is moving. At 20–30 miles per hour, the vehicle moves 9–13 m per second, so that, according to Equation (1), the mean RSSI of an AP within a few tens of meters changes by more than 1 dB between consecutive seconds, except near the point of closest approach, where the path loss is nearly flat. A smaller change is therefore more likely to reflect a stationary vehicle or the residual fluctuation of the average than motion relative to the AP. The threshold affects the number of retained samples rather than the set of retained APs: a larger value discards samples around the point of closest approach, which carry the most informative RSSI values, whereas a smaller value retains more samples from stationary periods, which bias the per-AP average toward a single distance. We also note that in a real multipath environment the RSSI does not always increase and decrease monotonically as the vehicle approaches and leaves an AP. Working on one-second averages rather than on individual samples smooths most of the fast fading, and the DBSCAN step removes the passes that do not follow the expected pattern. Samples that still deviate from the pattern are discarded by this filter, at the cost of a smaller sample count rather than of biased values. The number of APs and RSSI samples before and after data filtering and across different locations and months is reported in Table 1.

The filtering described above removes a considerable fraction of the BSSIDs and of the samples (Table 1). This is by design: the discarded APs are mostly those that were heard only briefly or only from a distance, whose RSSI is dominated by the path loss over an unknown distance rather than by the environment between the AP and the street. The retained APs are therefore biased toward those located along the driving route and received with a strong signal, which is also the population of APs that a deployed system would rely on, since the same filtering is applied to every run. Because identical criteria are applied to both months of each comparison, the filtering does not favor one leaf status over the other. The number of retained APs does, however, differ between months, as more APs become detectable once the leaves have fallen (Section 5.2).

## 5. Measurement Results and Analysis

**Figure 6:**
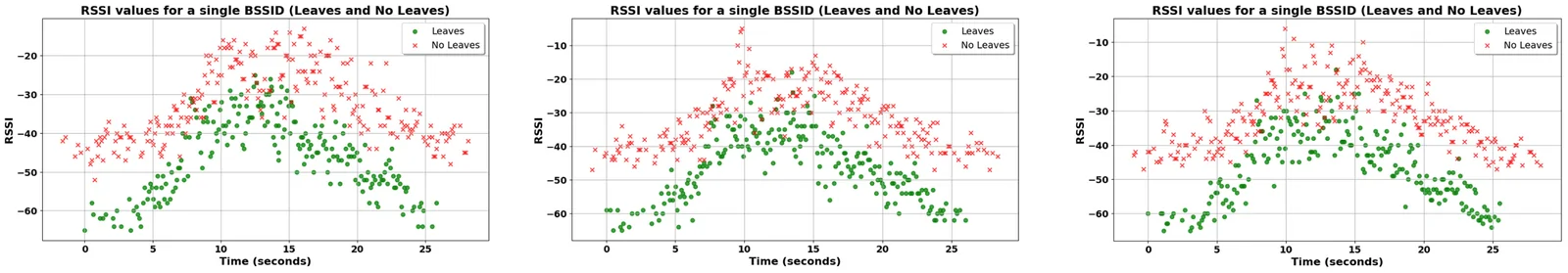
RSSI sample points of three access points in September (Leaves) and February (No Leaves) at Place B.

In this section, we aim to compare the data gathered across different months to evaluate the impact of leaves on RSSI values. The evaluation proceeds in four steps: the classification of the leaf status at three locations, including control experiments with unchanged foliage (Section 5.1), the reduction in the sensing rig to two channels (Section 5.2), the design for deployment in city blocks (Section 5.3), and the robustness of the measurements to the vehicle’s path and to the density of APs (Section 5.4). Classification accuracy, i.e., the fraction of correctly classified test samples, is used as the performance measure, since the task is a binary classification with balanced classes, and the Dummy Classifier provides the chance level against which it is compared. In Figure 6, the RSSI values of the same three BSSIDs captured in September and February at the same place are presented as an example to illustrate the data. The figure shows that, in general, the RSSI values captured in February are higher than those in September, suggesting that the absence of leaves in winter leads to stronger signal strengths. This observation indicates that the presence of leaves has a noticeable effect on RSSI, encouraging us to conduct further evaluations using machine learning-based classification methods to assess this relationship in greater detail.

### 5.1. RSSI-Based Classification of Leaf Status

To evaluate the impact of the presence or absence of leaves on RSSI values, we conducted a series of experiments across different months and three distinct locations—Place A, Place B, and Place C, as shown in Figure 3. Data collection was scheduled to capture the varying states of leaf density on the trees throughout the year. Specifically, at Place A, data were gathered in May, after the blooming of trees was complete and leaf density was at its peak, and in February, when leaf density is minimal. At Place B, data collection was conducted in September, during peak leaf density, and again in February. At Place C, representing a typical city block, data were collected weekly during the peak leaf-falling period from 1 October to 14 November. Our feature engineering process involved introducing a feature called “leaf-status” to the collected data, labeling the September, May and 1 October data as “Leaves” to indicate the presence of leaves and the February and 14 November data as “No Leaves” to signify their absence.

These labels were assigned on the basis of the measurement date together with a visual assessment of the trees along the route on the day of each run (see the photographs of Place C in Section 5.2). May and September correspond to a full canopy and February to bare trees. For Place C, 14 November was labeled “No Leaves” because, although a small number of leaves were still present on some trees, the large majority of the foliage had fallen, which is also the condition under which leaf collection is scheduled. The binary labeling is a simplification of a gradual process. The intermediate weeks (8 October to 8 November) were therefore not used for classification and are analyzed only through the trend of the mean RSSI in Section 5.2.

Additionally, to assess the accuracy of the classifier under unchanged leaf density conditions, we designed a second set of experiments focusing on short-term comparisons at the same locations. Specifically, at Place C, we analyzed data from 1 October against data from 8 October. Similarly, at Place B, RSSI measurements from one week in February were compared to data from another week within the same month. For clarity in classification, these data points were labeled as “Week1” and “Week2”. These experiments aim to evaluate the performance of the classifier in differentiating RSSI values within the same month, thereby ensuring that nonseasonal changes do not affect leaf density.

The primary goal of these experiments is to evaluate if RSSI values can be used to determine the seasonal changes accurately. To achieve this, we employed two widely used machine learning-based classification algorithms, Random Forest (RF) and Decision Tree (DT), for classification. These two classifiers were chosen for their suitability to the data: they operate directly on the RSSI features in dBm without scaling, require little parameter tuning, and are inexpensive enough to run on a Raspberry Pi. The Decision Tree offers a transparent model, and the Random Forest reduces its variance by averaging many trees. All processing was implemented in Python 3.9 using pandas 2.3, NumPy 2.0 and scikit-learn 1.6, whose DBSCAN, DecisionTreeClassifier and RandomForestClassifier implementations were used with their default parameters, except for 100 trees in the Random Forest. The captures were converted to CSV with tshark. During feature engineering, while the feature space can be extended to include additional features, such as the frequency of each BSSID, we focused on evaluating the effect of leaf density on RSSI by utilizing the RSSI values, the mean RSSI for each BSSID, and the mean RSSI for each channel as features. For a baseline comparison, we employed a Dummy Classifier (DC) that randomly predicted the “leaf-status”.

In all experiments, the training and test sets are drawn independently from the samples of each BSSID. The RSSI values of different BSSIDs differ widely, depending on the distance of the AP from the street, its transmit power and its placement, and together they cover a large range. In addition, since the distance between an AP and the sensing rig changes continuously as the vehicle passes, the samples of a single BSSID also span a wide range of RSSI values, from the first detection to the point of closest approach and back. Drawing both sets from the samples of every BSSID ensures that both of them cover this range, rather than, for instance, assigning the strong samples of an AP to one set and its weak samples to the other. Accordingly, 80% of the samples of each BSSID in each leaf-status class are used to train the classifier and the remaining 20% are used to test it. The per-BSSID and per-channel statistics used as features are computed from all samples of a run without using the labels, as they would be at deployment time, where the complete drive is available before classification. We note that this evaluation corresponds to the intended use of LeafSense, in which the same neighborhood, and hence largely the same set of APs, is revisited on every run and the classifier determines the leaf status from the RSSI of APs that it has observed before. The control experiments on two runs with unchanged foliage, which use the same split and features, confirm that the split itself does not yield accuracy above chance. The accuracies reported in Table 2 and Table 3 are therefore obtained with a sample-level split within each BSSID and characterize the repeated monitoring of a known neighborhood. They are not an estimate of the performance on independent measurement sessions, which would require further data collection in different blocks and cities.

The classification results based on RSSI across 2.4 GHz and 5 GHz frequency bands under varying and unchanged leaf density conditions are detailed in Table 2 and Table 3, respectively. These results confirm that the presence or absence of leaves clearly influences RSSI values. Generally, when the dataset includes an adequate number of BSSIDs and samples, the classification of “Leaves” and “No Leaves” achieves high accuracy. However, in the 2.4 GHz band at Place B, the limited dataset posed challenges in effectively training the classifier. Despite this, an 82% classification accuracy was still achieved, which is acceptable, especially compared to its counterpart in Table 3, which shows a 56% accuracy. In the 5 GHz band at Place C, the lower accuracy compared to other experiments might be due to the fact that, on 14 November, leaf density was not fully reduced, with some leaves remaining on the trees.

In addition, the results from Table 3 indicate that RSSI values are relatively stable when leaf density remains constant, which leads to low accuracy when classifying between “Week1” and “Week2” from the same month based on the captured RSSI data. These findings, coupled with the Dummy Classifier’s accuracy of approximately 50%—which is expected due to its random classification—serve as a contrast to highlight the efficacy of our analytical models. It is important to mention that, due to the complexity of the data gathering process, the experiment at Place A did not include data for channels 149 and 161 in May and channel 1 in February. In order to maintain consistency, the data for these channels was removed from the other run used for comparison. This resulted in a change in classification accuracy of less than 1%, indicating negligible effects.

The comparisons in Table 2 are made between runs that are several months apart, so factors other than the foliage may also differ between the two runs, such as temperature, humidity and precipitation, traffic, parked vehicles, or APs that were replaced or moved in the meantime. We addressed these factors in three ways. First, the areas were chosen such that no construction or infrastructure changes occurred between the runs, and all runs were made at the same driving speed with the rig in the same position in the car (Section 4.1). Second, the filtering of repeated passes and of stationary samples removes most of the effect of traffic and of the exact position of the vehicle (Section 4.3), and the robustness of the mean RSSI to variations in the route is evaluated in Section 5.4.1. Third, the control experiments in Table 3 compare two runs that are one week apart in the same leaf status. These runs are subject to the same day-to-day variations in weather, traffic and parked vehicles as the seasonal comparisons, yet the classifiers perform close to chance on them, which indicates that such variations are considerably smaller than the effect of the foliage.

The higher performance of the Decision Tree and Random Forest models in classifying the leaf status of trees based on RSSI underscores the capability of RSSI variations to effectively monitor and classify leaf status.

**Figure 7:**
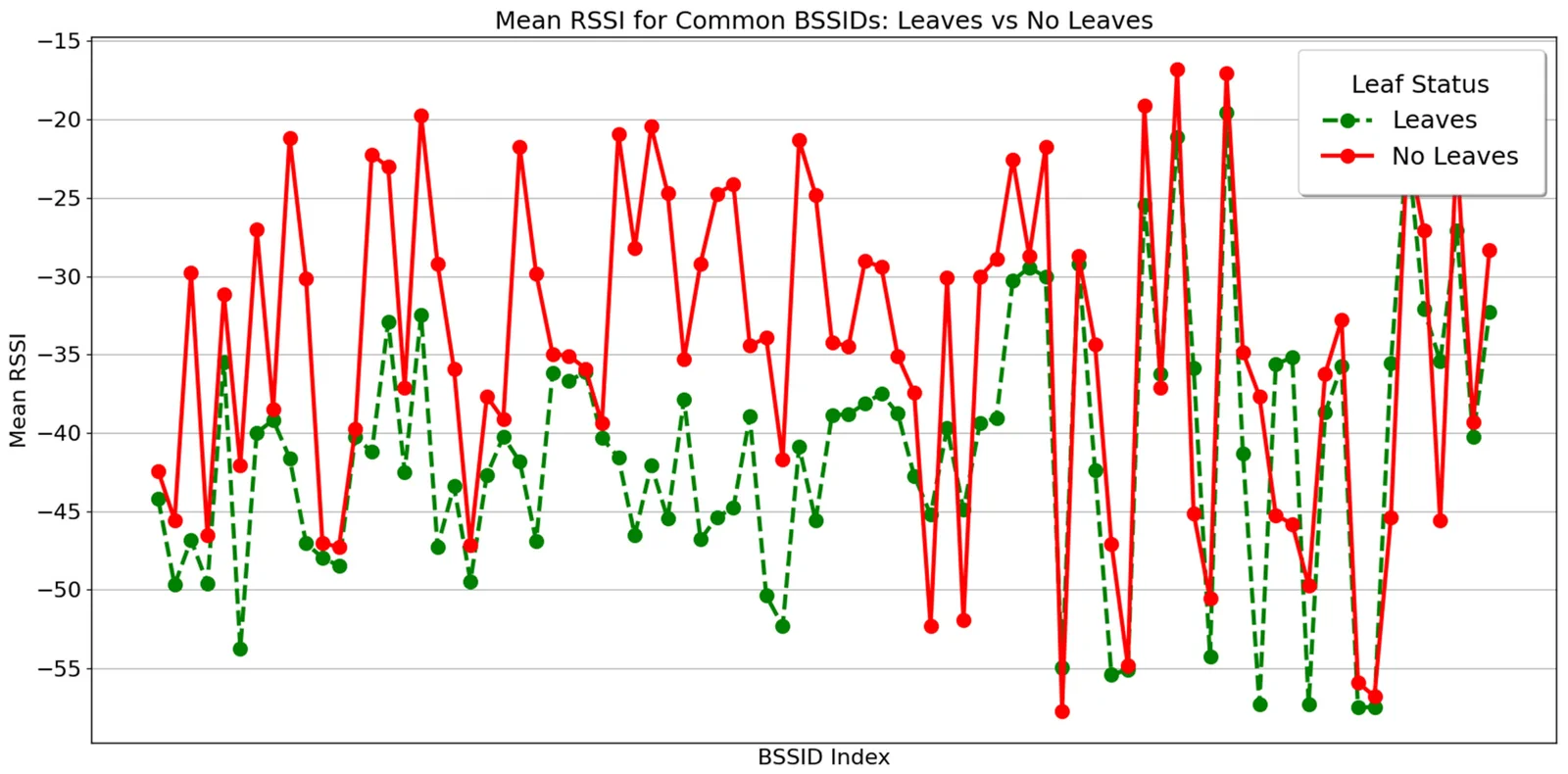
Mean RSSI for all common access points in September (Leaves) and February (No Leaves) at Place B.

Figure 7 displays the mean RSSI values for all common BSSIDs in our data collection runs at Place B during September and February, highlighting a clear difference between the mean RSSI values of September and February, thereby emphasizing the impact of seasonal changes on RSSI measurements.

### 5.2. Optimizing the Sensing Rig Design

So far, we have demonstrated that the RSSI values collected from 11 Wi-Fi channels can be used to effectively distinguish between the “Leaves” and “No Leaves” statuses. In this section, we aim to determine if we can reduce the number of channels without compromising classification accuracy. Simplifying the design of the sensing rig by reducing the number of channels, and consequently the number of Raspberry Pis, would make it more practical and efficient for real-world applications.

To explore this, we first need to ensure that sufficient data samples are available in a given location, even with fewer channels. To assess this, we focused on a representative city block, Place C (as shown in Figure 3), and conducted multiple data collection runs over several weeks during the peak leaf-falling period in October and November. To evaluate the coverage of each channel, we calculated the percentage of time (in seconds) where at least one data sample was collected for each channel. This percentage represents the proportion of seconds in which a channel had at least one sample, relative to the total duration of our data collection runs. The results for each channel, across different weeks, are presented in Table 4.

**Figure 8:**
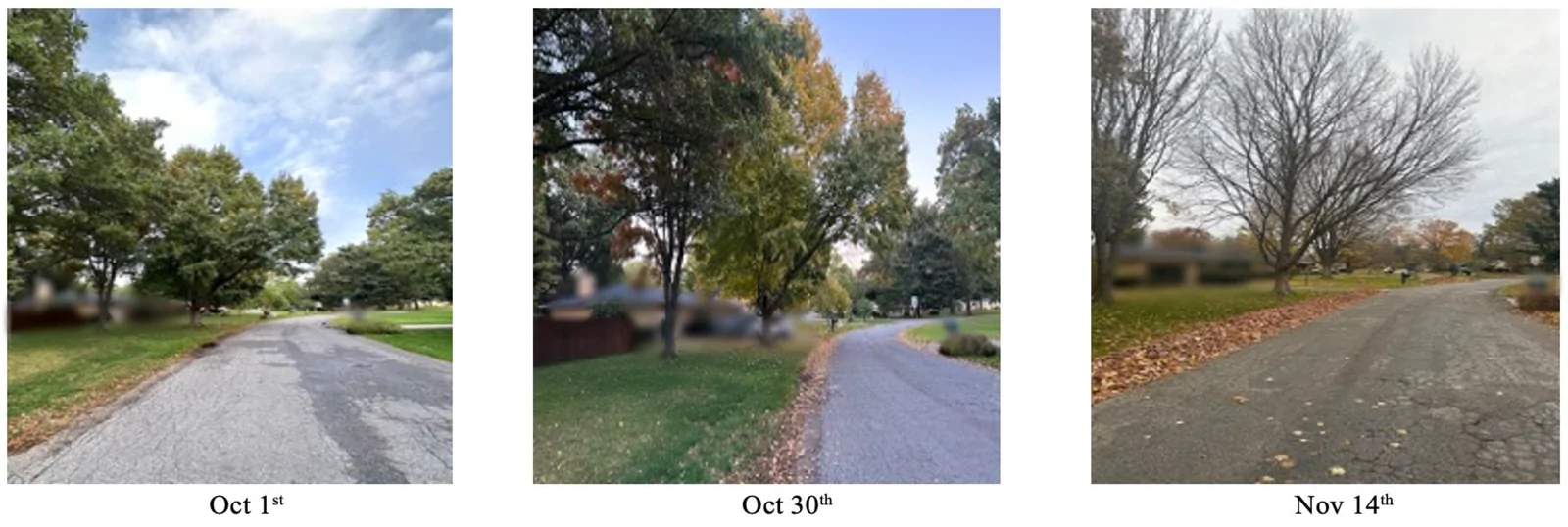
The leaf status at place C across different weeks.

**Figure 9:**
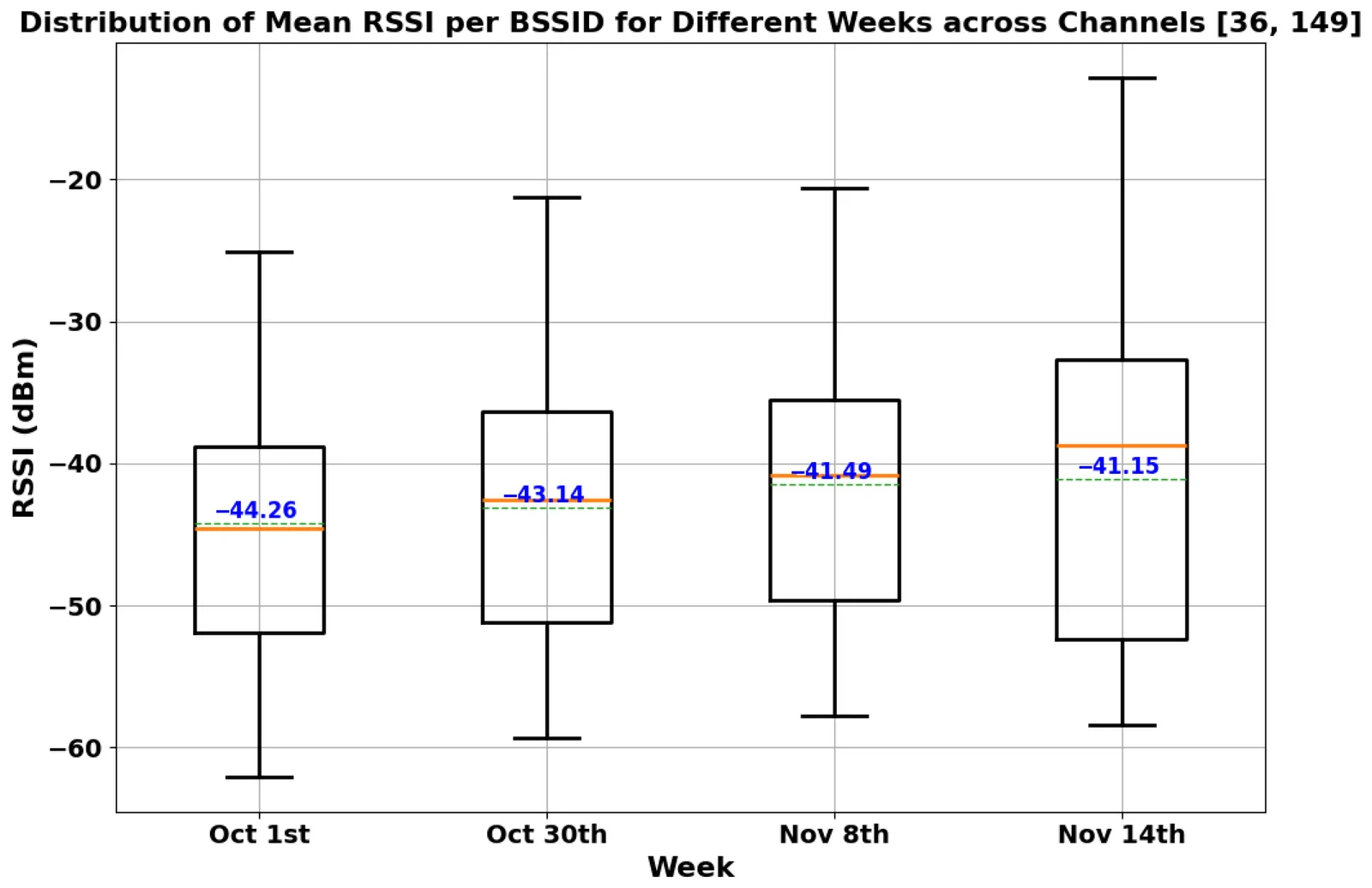
The mean RSSI distribution for Channels 36 and 149 across all BSSIDs. Boxes span the interquartile range of the per-BSSID mean RSSI and whiskers its full range; the orange line marks the median and the green dashed line the mean, whose value is given above it.

As shown in Table 4, the prevalence of Wi-Fi channels within the 5 GHz band is generally higher than those within the 2.4 GHz band. Among the 5 GHz channels, Channel 36 and Channel 149 have the highest prevalence. The combined data sample prevalence for these two channels across different weeks is reported in Table 5, showing that the prevalence percentage exceeds 90% in all weeks. In Figure 8, we illustrate the leaf status at the neighborhood where data was gathered across different weeks (the cars and houses in this image are blurred to ensure privacy), and in Figure 9, the mean RSSI distribution for Channels 36 and 149 across all BSSIDs is shown. This clearly demonstrates that, when the trees still have leaves (e.g., 1 October), the mean RSSI is lower (the overall mean RSSI is −44.26 dBm on 1 Oct). As the leaves begin to fall, the mean RSSI increases, with 14 November showing the highest mean RSSI (−41.15 dBm) and the least amount of leaves remaining. This shows the clear impact of leaf presence and absence on Wi-Fi signal strength.

Additionally, the number of captured BSSIDs increases from 1 October to 14 November, as reported in Table 6. This further supports the observation that leaves obstruct Wi-Fi signals, as more BSSIDs are detected once the leaves fall. The presence of leaves likely masks the signals from certain BSSIDs, making them undetectable until the leaves are gone.

Next, we evaluate the classification accuracy after optimizing the sensing rig by reducing the number of channels. We applied a similar approach to identify the best-performing channels in terms of prevalence at both Place A and Place B. The classification accuracy using only the selected channels is reported in Table 7. It can be seen that the accuracy achieved with just two channels is still high, and in some cases, exceeds that of using all channels. This is likely because some channels with lower prevalence have fewer data samples, which may correspond to locations without trees. These channels can skew the mean RSSI values, particularly across different leaf statuses, which is a crucial feature for classification. As a result, these channels may not accurately capture the impact of leaf presence or absence, reducing overall classification performance when they are included.

**Figure 10:**
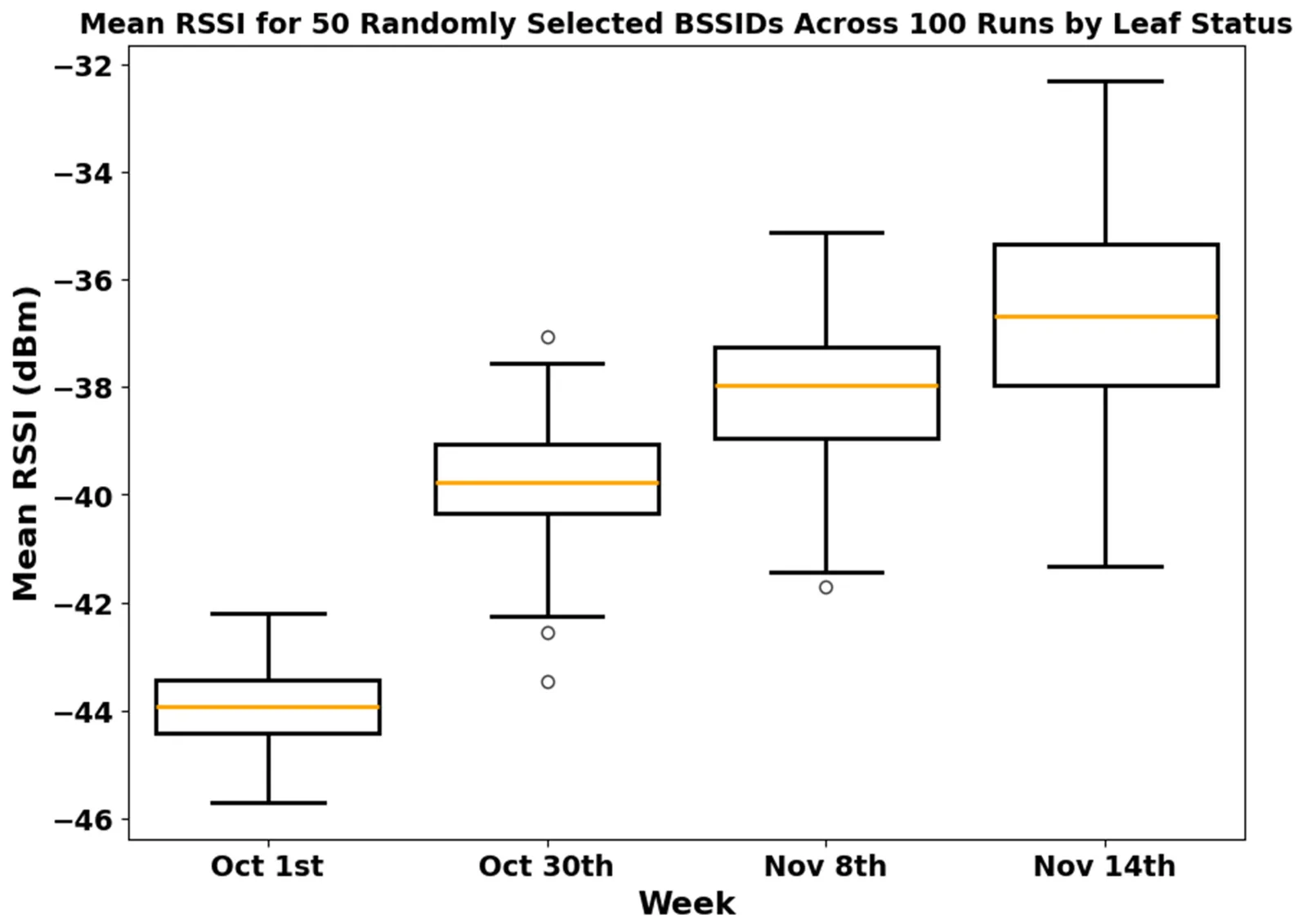
Mean RSSI for Channels 36 and 149 from 50 randomly selected BSSIDs at Place C across 100 runs.

To generate a Wi-Fi signature for each city block, we propose comparing the RSSI values of a consistent set of BSSIDs after each data collection run. To demonstrate the robustness of our approach against BSSID selection, we randomly selected 50 BSSIDs for each week, repeated this process 100 times, and then averaged the mean RSSI value for each week across all runs. Figure 10 illustrates the average RSSI values for Channels 36 and 149 from 50 randomly selected BSSIDs at Place C across 100 runs. By regularly comparing the RSSI values of these selected BSSIDs, monitoring their changes over time, and introducing a threshold for different leaf statuses, we can effectively assess the presence or absence of leaves in the area. Developing a more detailed algorithm to refine this comparison and enhance the accuracy of the leaf status detection will be a focus of our future work.

It is also noteworthy that, in certain cases, selecting only the single channel with the highest prevalence results in higher classification accuracy. One possible reason is that the difference between the RSSI values in different leaf status may be higher in certain channels. This larger difference enhances classification accuracy. However, to ensure our analysis is based on sufficient data, we opted to use two channels, as shown in Table 5, to maintain a prevalence of over 90% for all weeks. This choice balances data reliability with sensing rig optimization.

### 5.3. Dynamic Channel Switching for Leaf Monitoring in City Blocks

The ultimate goal of this work is to divide the city into distinct blocks and create a Wi-Fi signature for each block, which can be used as an indicator to monitor the presence or absence of leaves by tracking significant changes in RSSI values over time. By regularly observing the RSSI signature on a daily or weekly basis, we can assess the leaf status in each block. As demonstrated in the previous sections, RSSI values can effectively indicate the presence or absence of leaves. In Section 5.2, we further showed that collecting data from only two channels optimizes our sensing rig and yet yields reliable results.

However, as highlighted in Table 7, the optimal channels vary across different locations. In other words, the channels that provide the most reliable coverage differ from block to block. For this reason, it is crucial to collect data from the best-performing channels to ensure that the Wi-Fi signature in each block is constructed from reliable data. To achieve this, we propose an initial city-wide data collection using the main sensing rig (capturing all channels) to identify the two channels with the most coverage for each block, similar to what was done in our experiments at Place A, Place B, and Place C.

Once the best channels for each block are determined, the optimized sensing rig can be deployed for daily or weekly data collection—potentially mounted on a vehicle, such as a garbage collection truck. The sensing rig will need to switch between channels as it moves through different city blocks, given that each block may have different optimal channels. The timing of these switches can be guided by GPS data collected alongside the RSSI measurements. A critical factor to consider is the time required for the Raspberry Pi and external Wi-Fi adapters to switch between channels. To address this, we conducted an experiment in which the Raspberry Pi and a USB Wi-Fi adapter switched between channels while collecting and saving data. Our results showed that the time needed for this switch never exceeded 300 milliseconds, a negligible delay that does not disrupt the data collection process. It is worth mentioning that this experiment was conducted using a Raspberry Pi and an 802.11ac USB Wi-Fi adapter.

Therefore, for practical deployment, we can optimize the sensing rig to a single Raspberry Pi hosting two USB Wi-Fi adapters for the two selected channels and a GPS logger. This setup ensures efficient data collection while maintaining the flexibility needed to switch between channels as required by each city block.

### 5.4. Evaluation of Data Reliability and Access Point Density

#### 5.4.1. The Impact of Vehicle’s Path on RSSI Values

**Figure 11:**
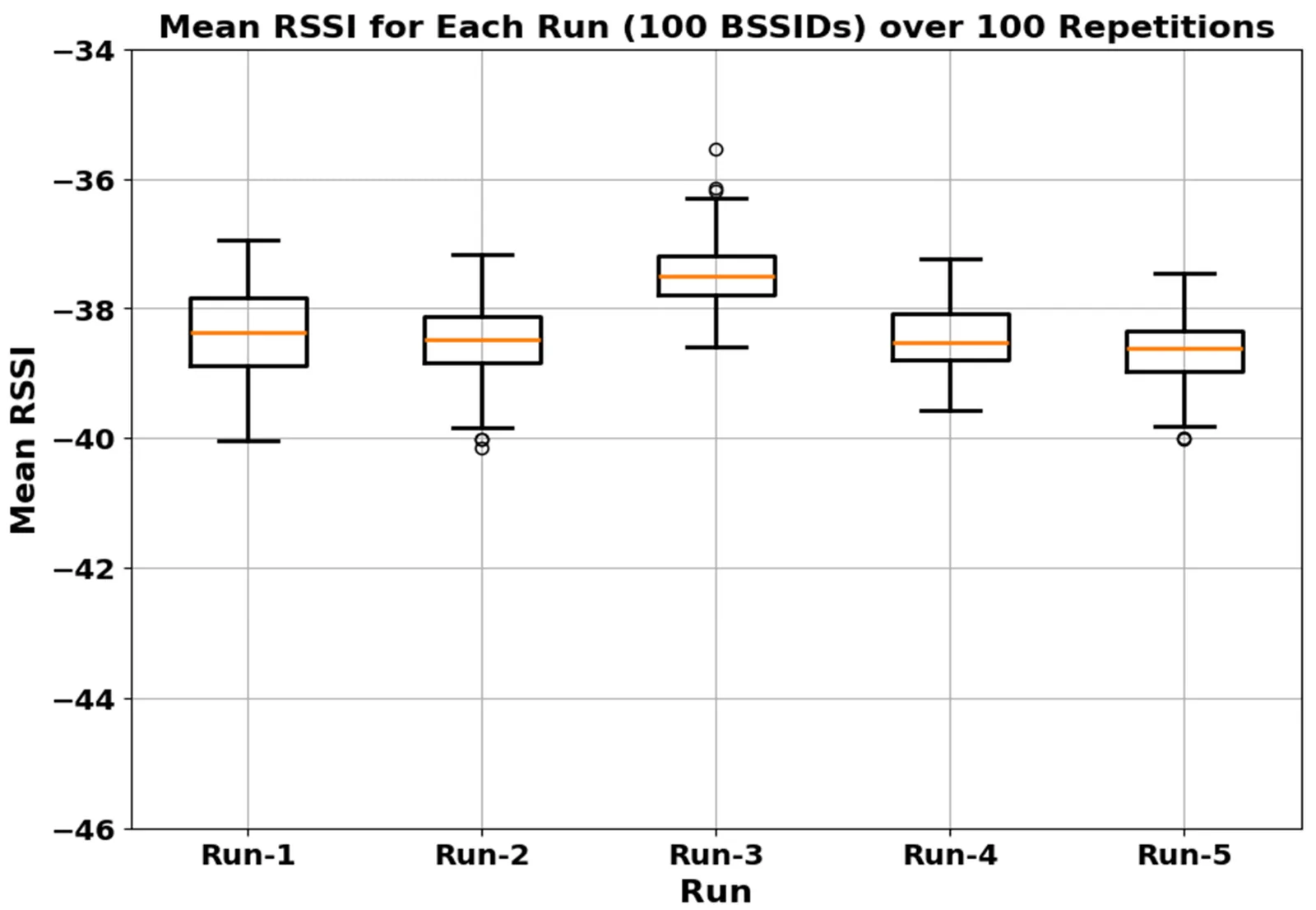
Mean RSSI from 100 randomly selected BSSIDs across 100 repetitions for 5 runs at Place C.

Since the sensing rig will be mounted on a vehicle, such as a garbage collection truck, there will be variability in the vehicle’s path due to obstacles, traffic, or parked cars. These variations in the route of the vehicle could affect the RSSI data collected. For example, the vehicle may deviate from its regular path to maneuver around a parked car, resulting in variations in the collected RSSI values. To evaluate the impact of such variations, five data collection runs at Place C (Figure 3) are conducted—three of these runs are in a clockwise direction and two are in a counterclockwise direction. The number of unique BSSIDs detected in each run after data filtering was 121, 120, 118, 128, and 122, respectively. For each run, 100 BSSIDs are randomly selected and the mean RSSI of these selected BSSIDs is computed. This process was repeated 100 times to ensure robustness. The results across all iterations are presented in Figure 11.

The results indicate that only minor variations are observed in the mean RSSI values across different runs. The consistency of the mean of the RSSI data across different runs suggests that it is robust to minor variations in the path. This shows that the reliability of the data remains unaffected by minor changes in the vehicle’s route, which suggests that, at least for a block of this size, a single run per visit is sufficient to obtain a representative mean RSSI.

#### 5.4.2. The Density of the Access Points

**Figure 12:**
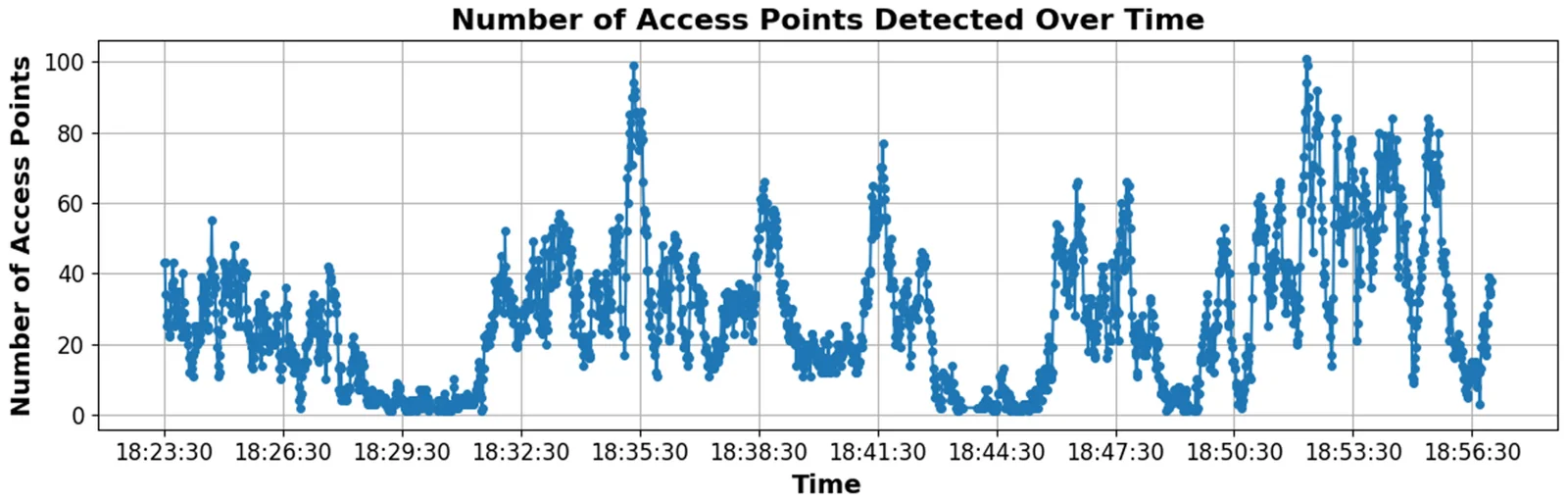
The number of access points per second.

To ensure that the number of access points is sufficient to provide reliable data samples across different areas of the city, we conducted a long-term data collection drive, covering various locations, including the downtown area. The drive lasted approximately 30 min, and the number of observed access points per second is shown in Figure 12. These results are obtained after applying data filtering.

We regard the number of access points as sufficient when at least 50 BSSIDs pass the filtering of Section 4.3 within a block, which is the number of BSSIDs used in Figure 10 to obtain a stable block signature. The figure shows that most areas of the city satisfy this condition. This supports our idea that, by mounting the sensing rig on a vehicle and driving across the city, a fair amount of data for meaningful analysis can be obtained. The figure shows nearly zero access points in two sections. Based on GPS data, these sections correspond to a bridge over a river that we crossed twice during the drive, approximately 15 min apart. Although this drive was designed to cover a variety of areas of the city within a single run, additional drives in other cities and in less densely built areas would help to confirm this observation more generally.

## 6. Conclusions

In this paper, we presented LeafSense, which leveraged Wi-Fi signals to monitor seasonal changes, specifically the falling of leaves in the autumn. The core idea of LeafSense is that the leaves introduce measurable reductions in RSSI, which can be used as an indicator of leaf presence or absence. The concept of LeafSense was evaluated over a significant corpus of data gathered over different seasons. The proposed processing pipeline eliminated data redundancy and we demonstrated that we could achieve classification results ranging from 81% to 94% with regard to the presence of leaves. Furthermore, we were able to show that even a minimal approach could achieve high accuracy with sensing only two 5 GHz channels. A control experiment on two weeks of unchanged foliage yielded near-chance accuracy, confirming that the classifiers respond to leaf status rather than incidental variation. We also found the results to be robust to small variations in the vehicle’s route, with AP density across the city sufficient for reliable sensing.

Although data were collected in three different urban areas and across several seasons, a larger number of locations and runs would allow the generalization of the results to be established more firmly, and extending the data collection to other cities and climates is a natural next step. The reported accuracies are likewise obtained with a sample-level split within each BSSID, and their evaluation on independent measurement sessions would require further data collection in different blocks and cities. The leaf status was treated as a binary label assigned per run. The weekly measurements at Place C show that the gradual fall of the leaves is reflected in the RSSI, which suggests that a finer-grained labeling of the canopy state, supported by ground truth, could further refine the detection. Finally, we tried to keep the conditions of the data collection runs as similar as possible, choosing areas without construction or infrastructure changes, driving at the same speed and keeping the sensing rig in the same position in the vehicle, and the control experiments indicate that the day-to-day variation in other factors, such as weather, traffic and parked vehicles, has a much smaller effect than the foliage, as the classifiers achieved only low accuracy when distinguishing two runs taken in different weeks with the same leaf status (Table 3). Incorporating such factors explicitly, for instance through weather data, could nevertheless improve the robustness of LeafSense over longer intervals.

One aspect of future work with respect to LeafSense includes constructing appropriate predictive models on a city block by city block basis, examining the granularity that is achievable. A second aspect includes examining the cost savings that could be achieved in truck dispatch logistics (leaf vacuum trucks). Beyond leaf monitoring, the same approach of tracking the RSSI of existing APs from a moving vehicle could be applied to other slow changes in the street environment, such as snow accumulation or vegetation growth, and to the monitoring of Wi-Fi coverage itself.

## Figures and Tables

**Table 1:** Number of BSSIDs and sample points before and after data filtering in different data collection locations.

		5 GHz				2.4 GHz			
Place	Month	BSSID Count		Sample Count		BSSID Count		Sample Count	
		Pre-Filtering	Post-Filtering	Pre-Filtering	Post-Filtering	Pre-Filtering	Post-Filtering	Pre-Filtering	Post-Filtering
A	May	949	527	74,185	30,818	247	50	6527	2161
	Feb	1072	543	86,381	33,793	100	33	2644	879
B	Sep	201	129	18,346	9733	48	12	981	389
	Feb	358	160	25,861	10,005	124	19	2292	613
C	Oct	210	158	39,421	13,396	69	24	4032	785
	Nov	331	252	70,426	25,019	131	65	8514	2442

**Table 2:** Classification accuracy of leaf status based on RSSI values across 2.4 GHz and 5 GHz bands under varying leaf density conditions using Random Forest (RF), Decision Tree (DT), and Dummy Classifier (DC).

	5 GHz			2.4 GHz		
Place	Random Forest	Decision Tree	Dummy Classifier	Random Forest	Decision Tree	Dummy Classifier
A (May/Feb)	83%	82%	50%	93%	93%	50%
B (Sep/Feb)	93%	94%	42%	81%	82%	52%
C (Oct/Nov)	82%	81%	50%	88%	88%	48%

**Table 3:** Classification accuracy of leaf status based on RSSI values across 2.4 GHz and 5 GHz bands under constant leaf density conditions using Random Forest (RF), Decision Tree (DT), and Dummy Classifier (DC).

	5 GHz			2.4 GHz		
Place	Random Forest	Decision Tree	Dummy Classifier	Random Forest	Decision Tree	Dummy Classifier
B (Feb/Feb)	55%	55%	50%	59%	56%	48%
C (Oct/Oct)	61%	61%	48%	60%	58%	52%

**Table 4:** The prevalence of each channel across different weeks at place C. The two channels with the highest prevalence in each week are shown in bold.

	Ch 1	Ch 6	Ch 11	Ch 36	Ch 40	Ch 44	Ch 48	Ch 149	Ch 153	Ch 157	Ch 161
1 Oct (%)	36.05	28.76	18.03	**89.00**	47.37	49.28	54.55	**77.03**	59.81	58.85	21.05
30 Oct (%)	20.17	25.21	10.08	**75.18**	23.38	58.99	69.78	**78.78**	49.64	64.39	18.35
8 Nov (%)	43.95	51.21	20.97	**80.76**	29.90	52.92	69.42	**84.88**	29.21	58.76	31.96
14 Nov (%)	64.63	10.48	31.88	**85.61**	28.77	55.09	71.58	**87.37**	35.44	52.98	33.33

**Table 5:** Combined coverage of channels 36 and 149 across different weeks at place C.

	1 Oct	30 Oct	8 Nov	14 Nov
Ch 36 & 149 coverage	93.31%	90.03%	92.09%	93.69%

**Table 6:** BSSID count for Channels 36 and 149, and all Channels across different weeks at place C.

BSSID Count	1 Oct	30 Oct	8 Nov	14 Nov
Ch 36 and 149	67	78	86	109
All Channels	182	209	251	284

**Table 7:** Selected channels and classification accuracy across different locations.

	Place A	Place B	Place C
Selected Channels	36 & 44	44 & 48	36 & 149
Classification Accuracy (%)	81	97	89

## Data Availability

The dataset and sensing rig design generated in this study will be made publicly available at https://github.com/SaeidMhrdd/leafsense-dataset (accessed on 19 October 2026). Anonymization includes location, BSSID, and SSID obfuscation through hashing and privacy-sensitive location transformations; no data frames were retained as part of the collection effort.

## Abbreviations

The following abbreviations are used in this manuscript:

AP	Access Point
BSSID	Basic Service Set Identifier
CSI	Channel State Information
CSV	Comma-Separated Values
DBSCAN	Density-Based Spatial Clustering of Applications with Noise
DC	Dummy Classifier
DT	Decision Tree
FTP	File Transfer Protocol
MIMO	Multiple-Input Multiple-Output
MQTT	Message Queuing Telemetry Transport
pcap	Packet Capture
RF	Random Forest
RSSI	Received Signal Strength Indicator
